# Electrocardiographic Imaging: A Comparison of Iterative Solvers

**DOI:** 10.3389/fphys.2021.620250

**Published:** 2021-02-03

**Authors:** Marta Borràs, Judit Chamorro-Servent

**Affiliations:** Department of Information and Communication Technologies, Universitat Pompeu Fabra, Barcelona, Spain

**Keywords:** inverse problem, ECGI, iterative methods, MFS, ART-SB, GMRES, RRGMRES, ART

## Abstract

Cardiac disease is a leading cause of morbidity and mortality in developed countries. Currently, non-invasive techniques that can identify patients at risk and provide accurate diagnosis and ablation guidance therapy are under development. One of these is electrocardiographic imaging (ECGI). In ECGI, the first step is to formulate a forward problem that relates the unknown potential sources on the cardiac surface to the measured body surface potentials. Then, the unknown potential sources on the cardiac surface are reconstructed through the solution of an inverse problem. Unfortunately, ECGI still lacks accuracy due to the underlying inverse problem being ill-posed, and this consequently imposes limitations on the understanding and treatment of many cardiac diseases. Therefore, it is necessary to improve the solution of the inverse problem. In this work, we transfer and adapt four inverse problem methods to the ECGI setting: algebraic reconstruction technique (ART), random ART, ART Split Bregman (ART-SB) and range restricted generalized minimal residual (RRGMRES) method. We test all these methods with data from the Experimental Data and Geometric Analysis Repository (EDGAR) and compare their solution with the recorded epicardial potentials provided by EDGAR and a generalized minimal residual (GMRES) iterative method computed solution. Activation maps are also computed and compared. The results show that ART achieved the most stable solutions and, for some datasets, returned the best reconstruction. Differences between the solutions derived from ART and random ART are almost negligible, and the accuracy of their solutions is followed by RRGMRES, ART-SB and finally the GMRES (which returned the worst reconstructions). The RRGMRES method provided the best reconstruction for some datasets but appeared to be less stable than ART when comparing different datasets. In conclusion, we show that the proposed methods (ART, random ART, and RRGMRES) improve the GMRES solution, which has been suggested as inverse problem solution for ECGI.

## Introduction

In Europe and North America, 50–100 sudden cardiac deaths per 100,000 people occur each year (John et al., 2012), and cardiac disease is a leading cause of morbidity and mortality (Wilkins et al., 2017; Roth et al., 2018). Non-invasive techniques to identify patients at risk, provide accurate diagnoses and perform ablation guidance therapy are currently under study (Shah, 2015). One of the most common non-invasive techniques is electrocardiographic imaging (ECGI; Wang et al., 2011). The ECGI technique enables study of the body surface potentials in relation to the heart anatomy. Heart activity is reconstructed from a dense array of body-surface electrocardiograms and patient-specific heart-torso geometry.

Despite the success of the ECGI technique in recent years (Ramanathan et al., 2004; Wang et al., 2011; Dubois et al., 2012; Haissaguerre et al., 2013; Cochet et al., 2014; Shah, 2015), improvements of the inverse problem solutions are needed to better understand and more precisely treat many cardiac diseases (Duchateau et al., 2019). The inverse problem is ill-posed, variations in heart surface potentials can lead to similar body surface potential distributions, and noise or artifacts in the measured data may negatively impact the solution. To ensure a unique and stable solution to this problem requires the use of: (i) regularization techniques (e.g., Tikhonov regularization) that can explicitly include a constraint or prior information in the minimization term, introducing a trade-off between the bias and the variance or (ii) iterative regularization algorithms that do not include functional regularization. The convergence of these algorithms to the solution is dependent on numerous iterations. Both regularization techniques and iterative regularization algorithms have been studied in the context of ECGI to seek a unique and stable solution to the problem described above (Guillem et al., 2020).

The main goal of this study is to apply methods from inverse problems in other fields, such as computer science and bioengineering, to the ECGI context. The aim is to adapt and test the ability of existing methods from other fields to reconstruct epicardial potentials. This study compares solutions in terms of amplitude, morphology and the resulting activation maps of the reconstructed ECGI electrograms.

The method of fundamental solution (MFS), a meshless method (Karageorghis and Fairweather, 1987) is applied to ECGI to solve the forward problem (Wang and Rudy, 2006). Iterative regularization algorithms are used to solve the inverse problem. Unlike Tikhonov regularization, iterative methods are not based on imposing constraints and therefore do not require *a priori* data, knowledge about the solution or the determination of a regularization parameter.

The forward problem has traditionally been reduced to cardiac and measurement surfaces by applying Green’s second theorem and discretized using the boundary element method (BEM; Barr et al., 1977). The MFS has been applied in this study to overcome the disadvantages of BEM, as the artifact introduced by the singularities adjacent to the surfaces in the fundamental basis function used and the high computational time required to mesh the heart and measurement surfaces (Wang and Rudy, 2006).

CardioInsight, a commercial implementation of the ECGI, uses MFS for the forward problem and uses spatial Tikhonov regularization or the generalized minimal residual (GMRES) method to reconstruct the epicardial potentials (Rudy et al., 2011). Tikhonov regularization seeks a balance between the bias and its variance, whereas the GMRES method is a projection iterative approach based on Krylov subspaces (Ramanathan et al., 2003; Rudy et al., 2011). In certain cases, the GMRES method has been found to localize potential features (e.g., multiple potential minima) that are lost in the Tikhonov solution and to improve the T-wave amplitudes (Ramanathan et al., 2003; Rudy et al., 2003). The GMRES is currently used to solve the MFS problem in the commercial CardioInsight technologies ECGI system (Rudy et al., 2011; Zeng et al., 2018). The performance of this technique for the MFS in a general setting was also shown (Karageorghis and Smyrlis, 2009).

Nevertheless, the GMRES method has proven to include the noise from the data. New methods based on “shifted” Krylov bases, such as range restricted generalized minimal residual (RRGMRES; Hansen, 2010), have been introduced in other fields (such as computed tomography) to combine “projection and regularization.” This has proven to reduce the noise introduced by GMRES (Hansen, 2010). However, these new methods have not yet been tested in ECGI.

This study approach began by testing the iterative GMRES, which has previously been tested in ECGI (Ramanathan et al., 2003; Rudy et al., 2003) and used with the MFS as forward problem. Second, the algebraic reconstruction technique (ART; Chamorro-Servent et al., 2013; Hansen and Jørgensen, 2018) – a Kaczmarz method widely used in tomographic imaging reconstruction, including cardiac images (Ziegler et al., 2009) – was adapted and tested. ART has not previously been used to reconstruct cardiac potentials and activation maps using the ECGI system. The next step was to test an ART method that combined a denoising at each iteration; this method has either not previously been used in a cardiac setting (Chamorro-Servent et al., 2013). Finally, we also applied the RRGMRES. The RRGMRES is a regularizing iterative method that was developed in the inverse problem field to improve GMRES accuracy for problems that include noise and/or artifacts in the data (Hansen, 2010), which is often the case for ECGI data.

All methods were validated using real data from the Experimental Data and Geometric Analysis Repository (EDGAR; Aras et al., 2015), which is an Internet-based open-source archive of curated data that is freely distributed to the international research community as a tool for application and validation of ECGI techniques. The objective of EDGAR is to facilitate collaboration among the ECGI research community to expedite the development and improvement of ECGI methods.

## Materials and Methods

### Forward Problem

In the MFS (Wang and Rudy, 2006), the electrical potentials can be expressed as a linear combination of the Laplace fundamental solutions over a discrete set of virtual source points. The latter located outside the domain of interest (Ω). In the ECGI setting, Ω is defined by the volume conductor enclosed by the body surface (Γ_*T*_) and the epicardial surface (Γ_*E*_).

Therefore, the potentials Φ are expressed as $\Phi \left(x\right)=a_{0}+\sum_{j=1}^{N_{S}}f\left(x,y_{j}\right)a_{j,}$ where *x* are the location points in the domain of interest (*x* ∈ Ω), (*y*_*j*_)_*j* = 1..*N*_*S*__ are the *N*_*S*_ fixed locations of the virtual sources points (*y*_*j*_∉Ω), and (*a*_*j*_)_*j* = 1..*N*_*S*__ are their respective coefficients. Here, *f* stands for the Laplace fundamental solution, which is defined as $f\left(x,y_{j}\right)=\frac{1}{4\pi r}$, where *r* = |*x*−*y*_*j*_| is computed as the Euclidean distance in 3D.

**FIGURE 1:**
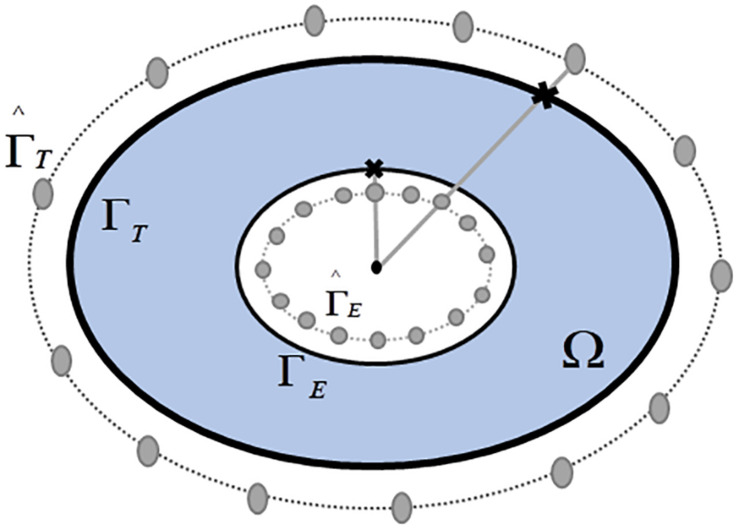
Schematic configuration of the pseudo-boundaries [deflating 0.8 and inflating 1.2, using the experimental values accorded in Wang and Rudy (2006)]. Where Γ_*T*_ is the body surface, Γ_*E*_ the epicardial surface, $\hat{\Gamma_{T}}$ is the virtual inflated body surface, $\hat{\Gamma_{E}}$ the virtual deflated epicardial surface, and **Ω** the domain of interest.

The virtual sources locations are fixed following (Wang and Rudy, 2006) such as in Figure 1. They are located by deflating the ${\left(x_{i}^{E}\right)}_{i=1,2,\cdots ,N_{E}}$ points, where reconstructing the epicardial potentials, at Γ_*E*_ (by a numerical factor 0.8), and inflating the ${\left(x_{i}^{T}\right)}_{i=1,2,\cdots ,N_{T}}$ locations of electrodes at Γ_*T*_ (by a factor 1.2), both relative to the geometrical center of the heart. Hence, there are a total of *N*_*S*_ = *N*_*T*_ + *N*_*E*_ virtual source points fixed locations, where *N*_*T*_ is the number of torso virtual source points and *N*_*E*_, the number of epicardial ones. It is important to note that MFS does not involve using a mesh. The solution of the Laplace equation (physics of the problem) is done by only using the locations $x_{i}^{E}$ and $x_{i}^{T}$, as well as the respective virtual sources.

Following the potentials expression above for Φ (*x*), where *x* ∈ Ω, we can express the potentials on the epicardial surface (*x* ∈ Γ_*E*_), too. Hence, Φ_*E*_ can be written as:

(1) $$\Phi_{E}=a_{0}+\sum_{j=1}^{N_{S}}f\left(x,y_{j}\right)a_{j},x\in \Gamma_{E},y_{j}\in \hat{\Gamma_{T}}\cup \hat{\Gamma_{E}}$$

Then, we can find the unknown coefficients of the virtual sources (*a*_0_, *a*_1_, ⋯, *a*_*N*_*S*__), by imposing, in an equivalent weight, the Dirichlet (Φ = Φ_*T*_) and the zero-flux or homogeneous Neumann (∂_*n*_⁡Φ = 0) boundary conditions for *x* ∈ Γ_*T*_. Hence, by using the potential definition and the values of its normal derivatives, this yields to solve the linear system:

(2) $$\Phi \left(x_{i}^{T}\right)=a_{0}+\sum_{j=1}^{N_{S}}f\left(x_{i}^{T},y_{j}\right)a_{j}=\Phi_{T}$$

(3) $$\partial_{n}\Phi \left(x_{i}^{T}\right)=\sum_{j=1}^{N_{S}}\partial_{n_{i}}f\left(x_{i}^{T},y_{j}\right)a_{j}=0$$

where Φ_*T*_ = (Φ_*i*_)_*i* = 1,⋯,*N*_*T*__ are the electrical potentials recorded on the locations of the torso electrodes $\left({\left(x_{i}^{T}\right)}_{i=1,2,\cdots ,N_{T}}\right)$.

For simplicity, this linear system can be written in a matrix notation as *Ma* = *d*, being

(4) $$M=\left(\begin{array}{c} \begin{array}{ccc} 1 & f\left(x_{1}^{T},y_{1}\right)\;\cdots & f\left(x_{1}^{T},y_{N_{S}}\right)\\ \vdots & \ddots & \vdots \\ 1 & f\left(x_{N_{T}}^{T},y_{1}\right)\;\cdots & f\left(x_{N_{T}}^{T},y_{N_{S}}\right) \end{array}\\ \begin{array}{ccc} 0 & \partial_{n_{1}}f\left(x_{1}^{T},y_{1}\right)\;\cdots & \partial_{n_{1}}f\left(x_{1}^{T},y_{N_{S}}\right)\\ \vdots & \ddots & \vdots \\ 0 & \partial_{n_{N_{T}}}f\left(x_{N_{T}}^{T},y_{1}\right)\cdots & \partial_{n_{N_{T}}}f\left(x_{N_{T}}^{T},y_{N_{S}}\right) \end{array} \end{array}\right)$$

(5) $$a={\left(a_{0},a_{1},\cdots ,a_{N_{S}}\right)}^{T}\epsilon {\mathbb{R}}^{1+{\text{N}}_{\text{s}}}$$

and

(6) $$d=\left(\begin{array}{c} \Phi_{T}\\ 0 \end{array}\right)\epsilon {\mathbb{R}}^{2{\text{N}}_{\text{T}}}$$

And solving a least-squares optimization problem, we can find the unknown sources coefficients (*a*ϵ*ℝ*^1 + N_s_^). To solve this optimization problem, we can use a regularization or iterative regularization method. In this work we used iterative regularization methods, as we explain in section “Inverse Problem.”

Once the coefficient vector is obtained, we can finally solve the potentials at any location in the domain of interest (*x* ∈ Ω) by using the potentials expression above forΦ(*x*). Therefore, the epicardial surface potentials (Φ_*E*_), can be reconstructed following Eq. 1, when *x* ∈ Γ_*E*_.

### Inverse Problem

The iterative methods solve a linear system *Ax* = *b*, which in mathematical formulation would be expressed as *x* = *A*^−1^*b*, if *A* was invertible and not ill-conditioned. In our application, the linear system is defined, as explained in section “Forward Problem,” by *Ma* = *d*. However, for simplicity, in this section we used the general notation since some of the methods described here require a square matrix, and our MFS’ matrix is not square. Then, we explained how to choose *A* and *b* in each respective inverse problem method’s subsection. In all cases, we used *x* = *a* = (*a*_0_, *a*_1_, …, *a*_*N*_*s*__).

In general, iterative methods always start with an initial vector and produce a sequence of iterations x_1_, x_2_, x_3_. that converge to some solution. The methods used in this study are semiconvergent iterative methods. In the semiconvergent methods the first iterations tend to converge to a good approximation of the exact solution, but at some stage, they start to diverge and converge toward the perturbed naïve solution, *A*^−1^*b* (Hansen, 2010). Then, the stopping criterion for these iterative methods is based on choosing the iteration number that early stops the optimization. To stop the iterations at the optimum time is equivalent to a large-scale regularization method (Hansen, 2010). For each method, we verified that the number of iterations correspond to the first local minimum of the “spatial” relative error (RE) and the first local maximum for the correlation coefficient (CC), respectively. “Spatial” RE and CC are described in section “Statistical Analysis.”

#### GMRES

In mathematics, the GMRES is an iterative method for the numerical solution of a non-symmetric system of linear equations. The method approximates the solution of *Ax* = *b* by the vector in an order-*r* Krylov subspace (*x*_*n*_ ∈ *K*_*r*_) that minimizes the Euclidean norm of the residual *r*_*n*_ = *Ax*_*n*_ − *b* (Saad and Schultz, 1986). The Arnoldi iteration is used to find this vector (Saad and Schultz, 1986).

In linear algebra, the order-*r* Krylov subspace generated by an *n*-by-*n* matrix *A* and a vector *b* of dimension *n* is the linear subspace spanned by the images of *b* under the first ***r*** powers of *A* (starting from *A*^0^ = *I*) (Saad and Schultz, 1986), that is: *K*_*r*_ (*A*, *b*) = span{*b*, *Ab*, *A*^2^*b*, …, *A*^*r*−1^*b*}.

This method requires a square matrix as an input, then, the matrix *A* in this work was written as *A* = *M*′*M*, and the vector *b* as *b* = *M*′*d*.

In this work we compared the computed solution of GMRES (previously used in the ECGI setting) with the results of the different iterative methods. With this purpose, the GMRES was implemented following the GMRES function from MATLAB R2019b.

#### ART and Random ART

The ART (Hansen, 2010) uses an iterative technique to solve the large linear system *Ax* = *b*. This method is used for underdetermined and ill-posed linear systems (Hansen, 2010).

The ART method is called as a row action method since these are methods that sequentially involve one row at a time (Hansen, 2010; Hansen and Jørgensen, 2018). The *k*th iteration consists of an update of the current solution vector *x*^*k*^ by scanning through the rows of *A* as follows:

(7) $$x^{k\left(i\right)}=x^{k\left(i-1\right)}+\frac{b_{i}-a_{i}^{T}x^{k\left(i-1\right)}}{{||a_{i}||}_{2}^{2}}a_{i}\mathrm{for}i=1,\ldots ,m$$

where *b*_*i*_ is the *i*th component of the right-hand side *b*, $a_{i}^{T}$ is the *i*th row of the coefficient matrix (turned into a column vector) and *m* = *N*_*s*_ + 1 (the rows of the MFS’ matrix).

Finally, it is necessary to save the updated solution vector in the new iteration (*k*):

(8) $$x^{k+1}=x^{k\left(m\right)}$$

In ART, *A* corresponds to the MFS’ matrix (*M*) which maps the virtual sources *x* = *a* = (*a*_0_,*a*_1_,…,*a*_*N*_*s*__) to the initial data *b* = *d* (i.e., *b* = [Φ_*T*_, 0, …, 0]).

The ART method used in this work was adapted to the ECGI setting following the open-source code provided by (Hansen and Jørgensen, 2018).

To initialize ART, two main different ways are commonly used in other fields: by setting the first solution vector (*x*_0_) as a zero vector or as a random vector. Some studies proved that using a random vector as first solution vector for ART, converge earlier than initialize it with a zero vector (Hansen, 2010). Hence, both choices for the initial vector of ART were tested in this work, to compare them. We called random ART, the ART method using a random vector for *x*_0_.

#### ART Split Bregman

The ART-Split Bregman (ART-SB) is an algorithm (Chamorro-Servent et al., 2013) which combines a denoising with the solution of the ART method, at each iteration. The ART-SB method has been used in other fields such as optical tomography (Chamorro-Servent et al., 2013), but it had never been tested with ECGI data. The denoising algorithm is based in a technique mostly used for compressed sensing technique (and L1-norm solution) called Split Bregman (SB; Goldstein and Osher, 2009).

ART-Split Bregman is implemented using a two-step iteration to the same system as the one used by ART in section “ART and Random ART” (*Ax* = *b*):

• The first step corresponds to the minimization problem

(9) $$x^{k}=\min_{\tilde{x}}{||A\tilde{x}-b||}_{2}^{2}$$

solved by ART for each *k*th iteration.

• The second step corresponds to the denoising problem

(10) $$\tilde{x}=\min_{\tilde{x}}\mathrm{TV}\left(\tilde{x}\right)+\frac{\mu }{2}{||\tilde{x}-x^{k}||}_{2}^{2}$$

where μ = 0.1 (such in Chamorro-Servent et al., 2013) is the weighting parameter for the fidelity term $||\tilde{x}-x^{k}||2_{2}$ and ***TV*** (or Total Variation). Thus, the solution $\tilde{x}$ constitutes the estimate for the next ART iteration (*k* + *1*).

Here, we considered TV as a 3D anisotropic TV (computed by the L1-norm derivatives of $\tilde{x}$ in each spatial direction). The SB method allowed splitting the problem in two subproblems that are easier to solve. To this end, the unconstrained problem of Eq. 10 is transformed into an equivalent constrained problem by applying the Bregman iteration (Goldstein and Osher, 2009). And we solved the L1-norm derivatives of $\tilde{x}$ in each spatial direction separately by using shrinkage operators (Goldstein and Osher, 2009).

The ART-SB algorithm has been adapted to this setting by using an open access software (Chamorro-Servent et al., 2013).

#### RRGMRES

As mentioned in section “GMRES,” GMRES method is useful for solving an inverse problem designed for non-symmetric square matrices. However, the order-*r* Krylov subspace includes the noisy right-hand side *b* = *b*^*exact*^ + *e* and thus the noise component *e*. This is a big disadvantage of the previously described GMRES method. The GMRES solution, obtained as a linear combination of the vector *b* in the first subspace, is likely to include a large noise component in the first subspace that increases iteratively (Hansen, 2010), and negatively impacts the final solution.

The RRGMRES aims to solve this issue. The main difference with GMRES is that RRGMRES works with the “shifted” order-*r* Krylov subspace which starts with the vector *Ab*, i.e., *K*_*r*_ (*A*, *b*) = *span*{*Ab*, *A*^2^*b*, …, *A*^*r*^*b*}. The advantage of this subspace is that the noise components are now multiplied by *A* already in the first subspace, which is demonstrated to produce a smoothing effect (Hansen, 2010).

Hansen (2010) performed the RRGMRES instead of the GMRES to improve the inverse problem error provided by the GMRES method. The RRGMRES method used in this work was the one developed by the authors in (Hansen and Jørgensen, 2018).

Like GMRES, the RRGMRES requires a square matrix to solve the linear system *Ax* = *b*. Hence, the method was applied after multiplying by the transpose matrix *M*′, at both sides of the MFS’s system.

### Test Bed Based Experiment

Ten datasets, obtained from the EDGAR (Aras et al., 2015), provided by three different research groups, were used in this work to develop the forward method and to test all the proposed inverse problems algorithms. All of them provided simultaneous epicardial and body surface potentials, meshes of the involved geometries (i.e., the body surface and the epicardium) and/or the electrode meshes were the potentials were measured. Therefore, to facilitate the comparative explained in section “Comparative Analysis,” we used the nodes of the provided meshes as the respective location points on the surfaces required to compute our MFS meshless. Torso and epicardial channels in which signals were absent or contained *NaN* numbers were discarded.

#### Dog Torso and Epicardial Recordings With Pacing (Maastricht 2015)

This dataset was provided by Maastricht University (Cluitmans et al., 2014). The data measured is a normal sinus beat and a paced beat (paced from the epicardial left ventricular apex). Body surface potentials and heart potentials were acquired simultaneously in a uniquely instrumented, anesthetized, normal dog.

The body surface potentials were recorded with *N*_*T*_ = 135 electrodes attached to the dog’s body surface, while the epicardial potentials were recorded with *N*_*E*_ = 83 electrodes implanted around the heart surface (“sock”). However, in the case of the paced beat, there were defective electrodes, and the remaining electrodes were placed in *N*_*E*_ = 65 locations.

#### Pig Torso, Epicardial Recordings With Pacing (Auckland)

This dataset was made available by the Auckland Bioengineering Institute at the Auckland University in 2015 (Bear et al., 2015). Similarly, as in “Dog Torso and Epicardial Recordings With Pacing (Maastricht-15-09-06),” simultaneous torso and cardiac surface recordings were measured concurrently in a pig during sinus rhythm and pacing rhythm, paced from the epicardial left ventricular apex.

In this case, they used *N*_*T*_ = 135 electrodes, attached to the pig’s torso, to record the body surface potentials, and *N*_*E*_ = 239 electrodes fixed to the inner surface of the “sock”, to record the epicardial potentials.

#### Ischemia Torso Tank With Epicardial Recordings (Utah 2015-05-02)

These experiments were performed by the Cardiovascular Research and Training Institute (CVRTI) and the Scientific Computing and Imaging (SCI) Institute at the University of Utah (Aras et al., 2015; Tate et al., 2018).

They used a cage to measure the epicardial potentials with *N*_*E*_ = 599 and a torso tank with *N*_*T*_ = 192 electrodes to record torso signals for this ischemia study.

### Comparative Analysis

#### Statistical Analysis

After reconstructing the epicardial potentials, the CCs and the relative root-mean squared errors –referred in this work as RE, were computed over the provided time steps following:

(11) $$\mathrm{CC}=\frac{\sum_{i=1}^{N}\left(\Phi_{{\mathrm{TE}}_{i}}-\bar{\Phi_{{\mathrm{TE}}_{i}}}\right)\left(\Phi_{{\mathrm{CE}}_{i}}-\bar{\Phi_{{\mathrm{CE}}_{i}}}\right)}{\sqrt{\sum_{i=1}^{N}{\left(\Phi_{{\mathrm{TE}}_{i}}-\bar{\Phi_{{\mathrm{TE}}_{i}}}\right)}^{2}}\sqrt{\sum_{i=1}^{N}{\left(\Phi_{{\mathrm{CE}}_{i}}-\bar{\Phi_{{\mathrm{CE}}_{i}}}\right)}^{2}}}$$

(12) $$\mathrm{RE}=\sqrt{\frac{\sum_{i=1}^{N}{\left(\Phi_{{\mathrm{CE}}_{i}}-\Phi_{{\mathrm{TE}}_{i}}\right)}^{2}}{\sum_{i=1}^{N}{\left(\Phi_{{\mathrm{TE}}_{i}}\right)}^{2}}}$$

where Φ_*TE*_ were the target potentials, i.e., the epicardial potentials recorded by the sock’s (or cage’s) electrodes provided by EDGAR (in each case), and the computed potentials, Φ_*CE*_, were the reconstructed ones.

Following this, we did two studies: (i) Considering both potentials (target and computed) at each spatial locations, averaged over time (*N*); (ii) Considering both potentials at each time, averaged over space (*N*). In the first study we aimed to provide a “spatial” visualization and in the second study, a “temporal” one. CCs and RE have been largely used to compare electrical potentials in the ECGI setting (Cluitmans et al., 2018). The highest CC represents the best morphology and the lowest RE the best amplitude of the reconstructed potentials.

**FIGURE 2:**
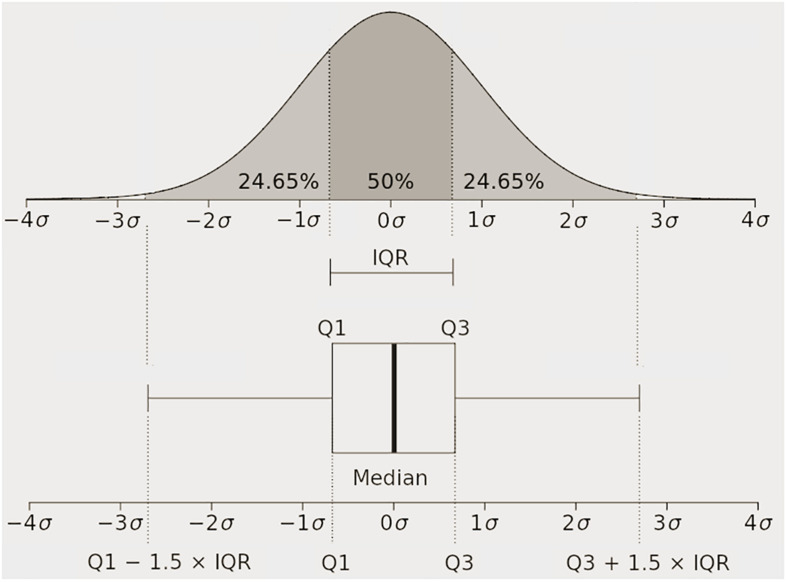
Quartiles (Q_*_) and boxplots’ description schema.

For the “spatial” visualization, we plotted the Q1, median (or Q2) and Q3, and the lines extending from the boxes (whiskers) indicating the variability outside Q1 and Q3 following the Figure 2. Additionally, the “^∗^” depicts the outliers. An outlier represents an observation that is numerically distant from the rest of the data (i.e., located outside the whiskers of the boxplot). Our boxplot draws points as outlier if they are greater than Q_3_ + m_w_ (Q_3_ − Q_1_) or less than Q_1_ − m_w_(Q_3_ − Q_1_), where m_w_ is the largest whisker length.

For the temporal visualization, first we computed the root-mean-squared (RMS) potentials:

(13) $$\mathrm{RMS}=\sqrt{\frac{\sum_{i=1}^{N}{\left(\Phi_{{\mathrm{TE}}_{i}}\right)}^{2}}{N}}$$

where N is the number of spatial locations.

Afterward, we compared the CCs, and REs of the reconstructed potentials averaged over a 100 ms window around the QRS complex. This window is calculated based on the RMS following (Bear et al., 2015).

This can provide a quantitative comparative since variations on the QRS complex indicate issues on ventricular depolarization and on myocardial ischemia data (Burnes et al., 2000; Romero et al., 2016; Almer et al., 2019).

#### Activation Maps

Finally, to see the differences of the methods for the different heart surface’s regions, we computed the recorded and ECGI-reconstructed dV/dT patterns when possible. These patterns were first computed for the over activation and recovery time, and afterward over the QRS complex (activations maps).

The locations of the dV/dT patterns over activation and recovery time were depicted with the corresponding “spatial” CC and RE (following section “Statistical Analysis”). We showed a percentage of accurate points by selecting a fixed threshold (CCs > 0.8 and REs < 0.5, both chosen experimentally for the specific datasets used in this written work), as the figures show in the next section.

## Results

We computed the reconstruction of the 10 datasets introduced in section “Test Bed Based Experiment.” Those include: two paced rhythms against two sinus rhythms, and four myocardial ischemia datasets against two control datasets. The main results of the comparative study described in section “Comparative Analysis” can be found in the next subsections.

### Boxplots of the “spatial” CC and RE Averaged Over Time of the Reconstructed Potentials

**FIGURE 3:**
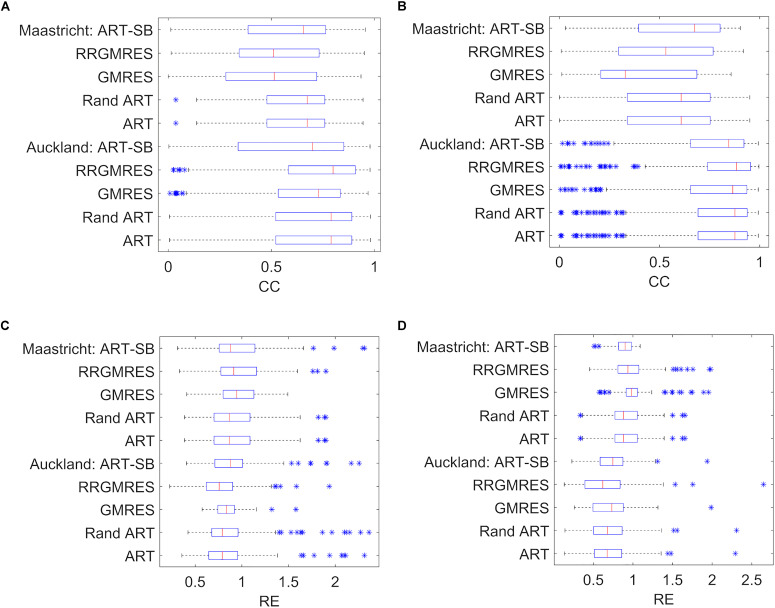
Boxplots showing the mean, the distribution, the standard deviation (“whiskers”) and the outliers (“*”), for: **(A)** the CC results of the sinus rhythm; **(B)** the CC results of the paced rhythm; **(C)** the RE results of the sinus rhythm; **(D)** the RE results of the paced rhythm. *Y*-axis specify the dataset and the reconstruction method used.Similarly, for a two control subjects and four ischemia ones from the Utah dataset, the boxplots to compare the reconstructions with the respective target potentials (i.e., the provided potentials measured at the cage) were respectively shown in Figures 4, 5.

**FIGURE 4:**
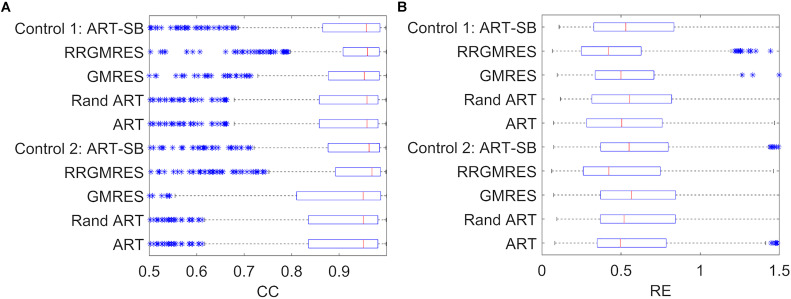
Boxplots showing the mean, the distribution, the standard deviation (“whiskers”) and the outliers (“*”), for: **(A)** CC and **(B)** RE. The *y*-axis show the reconstruction method used for each one of the two control recordings of the Utah dataset (Control 1, Control 2).

**FIGURE 5:**
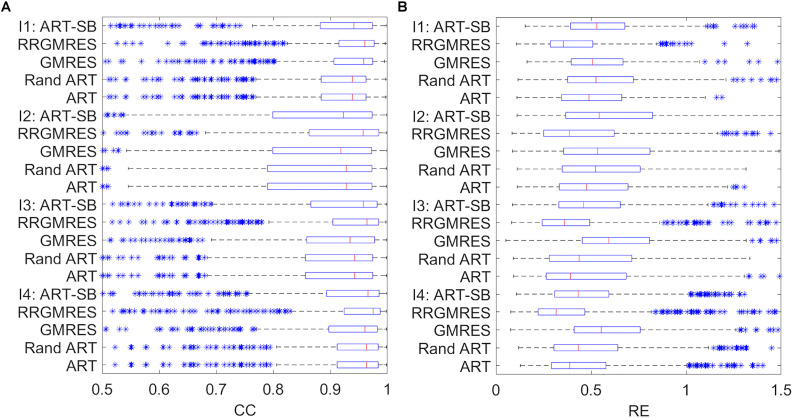
Boxplots showing the mean, the distribution, the standard deviation (“whiskers”) and the outliers (“*”), for: **(A)** CC and **(B)** RE. *Y*-axis show the reconstruction method used for each one of the four ischemia recordings of the Utah dataset (I1, I2, I3, I4).

We plotted the boxplots following the specifications in section “Statistical Analysis,” for the different quartiles of the resulted CCs and REs for each reconstructed dataset. Boxplot of the averaged CCs and REs over time for each space location can be found in Figures 3–5. We refer to them as “spatial” CCs and REs. Additionally, we represented the outliers by “^∗^.”

For the Maastricht and Auckland datasets, the boxplots to compare the reconstructions with the respective target potentials (i.e., provided potentials measured at the sock) were depicted in Figure 3.

### “Temporal” CC and RE Averaged Over Space of the Reconstructed Potentials During a Defined Temporal Window Over QRS

Following section “Statistical Analysis,” the CCs and REs were averaged over space for each time of a 100 ms window around the QRS complex. We refer to them as “temporal” CCs and REs.

**FIGURE 6:**
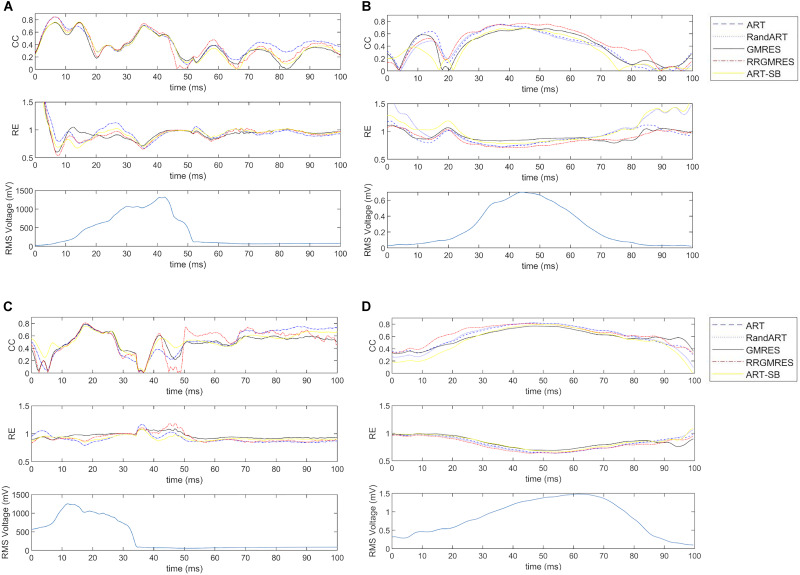
For the datasets: **(A)** the sinus rhythm of Maastricth; **(B)** the sinus rhythm of Auckland; **(C)** the paced rhythm of Maastricth; **(D)** the paced rhythm of Auckland. From top to down: “temporal” CC and RE of each reconstruction technique and RMS of the target potentials. All of them for a defined 100 ms window over the QRS.

For the Maastricht and Auckland datasets, “temporal” CCs and REs of the reconstructions compared with the respective target potentials (i.e., provided potentials measured at the sock) were depicted in Figure 6, together with the computed RMS.

**FIGURE 7:**
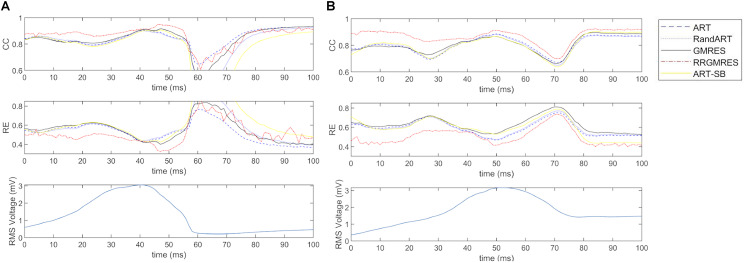
RE and CC of each reconstruction technique and RMS of the target potentials, averaged over space during a defined temporal window over the QRS, for **(A)** a control Utah dataset (Control 1) and **(B)** an ischemia Utah dataset (I3).

Similarly, for the Utah dataset, “temporal” CCs and REs of the reconstructions compared with the respective target potentials (i.e., the provided potentials measured at the cage) were shown in Figure 7, together with the computed RMS.

### Activation Maps

**FIGURE 8:**
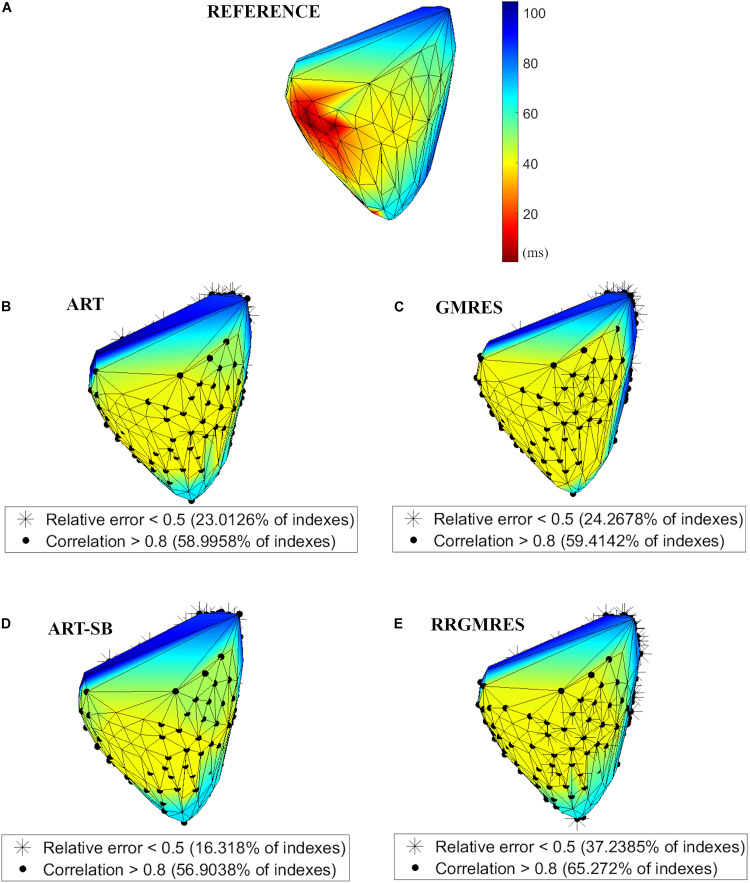
Activation maps applied to the paced rhythm Auckland dataset, for: **(A)** Reference potentials; **(B)** ART; **(C)** GMRES; **(D)** ART Split Bregman; **(E)** RRGMRES. All figures have the same colorbar than panel **(A)**. For each activation map, the locations corresponding to the “spatial” RE < 0.5 and CC > 0.8 are overplotted as shown in the legend.

**FIGURE 9:**
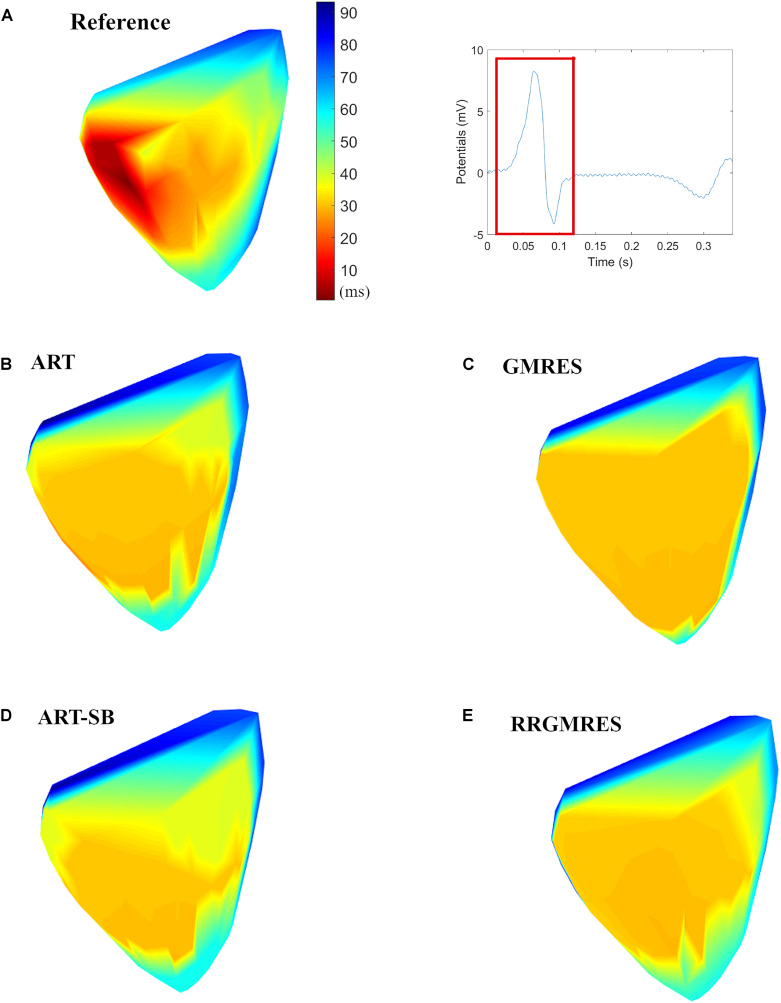
Activation maps of the potentials during a 100 ms window (over QRS), applied to the paced rhythm Auckland dataset for: **(A)** Reference potentials and QRS window; **(B)** ART; **(C)** GMRES; **(D)** ART Split Bregman; **(E)** RRGMRES. All figures have the same colorbar than panel **(A)**.

For demonstration purposes, in this subsection, we showed a view of the resulted activation maps for the different reconstruction methods against the target one for the paced rhythm Auckland dataset: Figure 8 shows the dV/dT pattern over activation and recovery time, and Figure 9 over the QRS complex (activations maps).

As mentioned in section “Pig Torso, Epicardial Recordings With Pacing (Auckland-2012-06-05)” the pacing was done in the epicardial left ventricle apex.

Additionally, in Figure 8 we outlined the locations with the best “spatial” CC and RE following the explanation in “Activation Maps.”

The reader can refer to the Supplementary Material of this manuscript to find the activation maps of the other sinus and paced rhythm datasets used in this work.

## Discussion

The aim of this work was to adapt and apply four different inverse problem iterative methods from computer science and other bioengineering applications to the field of ECGI. The solutions of these methods were compared to the respective target potentials (i.e., provided epicardial potentials measured with sock or cage) and the computed GMRES solution. The GMRES method is used in the commercial ECGI system CardioInsight, which was introduced in the European Union in 2011 and in the United States in 2015 (Rudy et al., 2003). This method has proven to overcome some limitations of Tikhonov regularization (Ramanathan et al., 2003; Rudy et al., 2011; Zeng et al., 2018). The CardioInsight system uses the MFS method as forward problem (Rudy et al., 2011; Zeng et al., 2018), and this method was also used in the present study.

The analyzed iterative inverse problem methods are semiconvergent (see section “Inverse problem”). Codes that guaranteed convergence were used to solve the different iterative methods. Based on the analysis and verification process about the number of iterations (see section “Statistical Analysis”), 500 iterations were used for ART, random ART and ART-SB; 10 iterations were used for GMRES; and 100 iterations were used for RRGMRES (results not shown).

As explained in section “Comparative Analysis” of this paper, the reconstructed potentials were compared with the target measures via: (i) boxplots that displayed the amplitude and morphology of the reconstructed epicardial potentials for each spatial point averaged over time (referred as “spatial” CC and RE); (ii) RE and CC during a defined temporal window over the QRS onset (referred as “temporal” CC and RE); and (iii) activation maps if the provided geometries rendered their use possible. As previously mentioned, the lowest RE value indicates the best amplitude for the reconstructed potentials/activation maps, and the highest CC value indicates the best provided morphology.

The boxplots of the “spatial” REs and CCs for the reconstructed epicardial potentials of the various datasets show the median, distribution, deviation, and outliers (see Figure 2). In terms of REs and CCs, the ART, random ART, and RRGMRES methods had the best results, whereas the GMRES reconstructions had the worst. Specifically, the GMRES method resulted in median and Q3 results that were worse than those of the three other methods. In the case of Auckland dataset, the reconstructions of the recordings during the paced rhythm (see Figures 3B,D) had better results than those during the normal sinus rhythm for both CC and RE (Figures 3A,C). In Maastricht dataset, no conclusive differences between the CC of the two rhythms were observed, regardless of GMRES technique. However, the paced rhythm provided worse results in RE than the sinus one. The solutions provided by the ART and random ART methods were among the best results with regard to stability between the paces and sinus rhythm datasets.

In the “spatial” analysis of ischemia and control datasets from Utah (Figures 4, 5), the boxplots showed that each method achieved a similar “spatial” CC, as well as a good median and distribution. The control datasets (Figure 4) provided similar results in that the RRGMRES and ART methods had slightly better results than the GMRES method. Similar behavior was observed in the ischemia and control datasets (Figures 4, 5); specifically, RRGMRES was the best reconstruction method, followed by ART, random ART, ART-SB, and finally GMRES. The results of the different methods had a higher statistical significance regarding “spatial” RE than CC, mainly for GMRES (Figure 5).

In Figure 6, the “temporal” CC and RE were displayed (over a time window of 100 ms around the QRS complex) for the Maastricht and Auckland datasets. In all cases, the best reconstructions (i.e., those with the lowest RE and highest CC) were observed during the QRS complex. The ART, Random ART and RRGMRES methods achieved the best reconstructions during the QRS for the Maastricht dataset. Differences between the reconstruction techniques were almost inappreciable in the QRS complex; however, GMRES was found to provide the worst reconstructions of all the methods examined. In the Maastricht dataset, the main differences were in the RE. However, the values of “temporal” CC and RE were not dependent on the rhythm.

The “temporal” CC and RE were also analyzed over a time window around the QRS complex for the Utah datasets. Figure 7 displays the results for one control and ischemia datasets. During the period out of QRS, unstable behavior and poor values were observed for the CC and RE solutions for all reconstruction methods, but ART proved to be the most stable and best method (not shown results). This unstable behavior out of the QRS could explain the higher number of outliers found in Figures 4, 5 against the Figure 3. During the QRS complex, the RRGMRES method achieved the best reconstruction through time for the ischemia dataset, although it was not smooth for the control dataset. ART also provided a good reconstruction for both ischemia and control datasets.

Activation maps were plotted based on the dV/dT. Figures 8, 9 show the activation maps of the paced rhythm for the reference and each reconstruction method of Auckland dataset. Figure 8 refers to both activation and recovery times, and Figure 9 only to activation times (QRS complex). Additional activation maps from other datasets are included in the Supplementary Material.

For each activation map of Figure 8, location points of the epicard were overplotted with the best reconstructed potentials regarding the “spatial” CCs and REs averaged over time, as explained in section “Statistical Analysis.” The locations of the reconstructed potentials with the best REs strongly corresponded to the atria (see Figure 8). However, the locations of the reconstructions with the best CCs corresponded to both atria and ventricles (see Figure 8). At the epicardial left ventricle apex (the location where the pacing was done), the RRGMRES method provided the most accurate reconstruction of the tested methods.

The activation map did not appropriately identify the transition between early and late activation, which may be due to the small amount of torso and heart locations that were used to reconstruct the solution or to issues with the high ill-posedness of the problem. Then, additional improvements are needed. However, the activation maps of Figure 9 suggest a better transition between early and late activation times for ART, ART-SB and RRGMRES than GMRES.

Epicardial potentials, Φ_*E*_, could be determined at more locations than the ones computed by applying Eq. 1 from section “Forward Problem” to additional *x*∈ Γ_*E*_ locations. This study’s aim was to compare the reconstructed solution with the measured epicardial potentials (target); therefore, to avoid introducing interpolation errors, the results were only compared in the epicardial electrode locations. This is a limitation to provide further clinical discussion on the results. However, the RRGMRES, ART, and random ART methods indicate an improvement in the ventricles and the QRS complex. These results suggest that these methods could provide clinical advantages over GMRES, due to the importance of the QRS in ventricular depolarization and the ischemia-related changes found in the QRS complex (Burnes et al., 2000; Romero et al., 2016; Almer et al., 2019).

The methods examined in this study improved the solution when treating the MFS as a forward problem. It is unknown whether the conclusion would differ if the forward problem was formulated with a mesh-based method. However, the study results clearly indicate that the RRGMRES method is an improvement over the GMRES method. This improvement is attributable to the noise and artifact reduction of RRGMRES in Krylov subspaces, as explained in section “RRGMRES.”

Another benefit of Krylov-based methods is their ability to recycle subspaces (Kilmer and De Sturler, 2006). This benefit could lead to important progress in reducing the computational burden of future clinical explorations.

In this work, we selected the necessary virtual sources for the MFS following (Wang and Rudy, 2006). However, different virtual source’s choice in the MFS modifies the ill-conditioning of the related inverse problem (Karageorghis and Smyrlis, 2009; Chamorro-Servent et al., 2016). And, when the distances of the virtual sources from the boundary are large, the ill-conditioning becomes more severe (Karageorghis and Smyrlis, 2009). Finally, it is well-known that the number of needed iterations for iterative solvers depends on the ill-conditioning of the problem (Hansen, 2010). GMRES’ solution is not affected by different distance’s choice of the virtual sources (Karageorghis and Smyrlis, 2009). GMRES and RRGMRES are both based on Krylov bases. Then, we do not expect either differences comparing the solutions provided for both methods. However, further study is needed about the affectation of these sources on the ART, random ART, and ART-SB solutions.

In summary, the results of this study indicate that the ART, random ART and RRGMRES methods yielded the best reconstruction of potentials, followed by ART-SB, and finally the GMRES method. The random ART method has traditionally been used to reduce the number of necessary iterations for the ART method. However, the ART method does not require computation of random vectors for each inverse problem, as the random ART method does. The ART method was identified as the best method, due to the negligible differences between the solutions of the ART and random ART methods and the number of iterations required.

The results also indicate that ART is the most stable method of solving both paced and sinus (Maastricht and Auckland) datasets. This method also provided a reliable solution for control Utah dataset. The RRGMRES method reconstructions for the Auckland and Utah ischemia’s datasets provided the best results of all methods examined. However, the RRGMRES method did not work well for all datasets (e.g., Maastricht dataset in Figure 3) or out of the QRS for the Utah datasets. In conclusion, the proposed ART, random ART and RRGMRES methods were all improvements on the GMRES, which is the only method that had previously been tested in ECGI (Ramanathan et al., 2003). Based on the findings of this study, ART proved to be the most stable method of those examined.

## Data Availability Statement

The raw data supporting the conclusions of this article will be made available by the authors, without undue reservation.

## Author Contributions

MB and JC-S conceived the presented idea. MB adapted the different inverse methods, did the statistical comparative, and developed all the results. Both authors discussed and contributed to the writing of the final manuscript.

## Conflict of Interest

The authors declare that the research was conducted in the absence of any commercial or financial relationships that could be construed as a potential conflict of interest.
