# Supplementary material for: Electrocardiographic Imaging: A Comparison of Iterative Solvers
Source: Front Physiol. 2021 Feb 3;12:620250. doi: 10.3389/fphys.2021.620250 (PMC7886787; doi:10.3389/fphys.2021.620250)
Supplement: Supplementary file 1 [file Data_Sheet_1.PDF]

Supplementary Material

1 Supplementary Data

In this supplementary material we show the missing activation maps for Auckland and Maastricht datasets when comparing with the computed activation maps from the respective provided measured sock potentials reference. Like in the section 3.3 of the related manuscript, we outlined the locations with the best CC and RE following the explanation in 2.4.

1.1 Supplementary activation maps for Auckland dataset

Supplementary Figure 1 shows the dV/dT pattern over activation and recovery time for Auckland dataset. Additionally, we outlined the locations with the best “spatial” CC and RE following the explanation in 2.4.2.

For the normal sinus Auckland dataset, RRGMRES achieved the best reconstructions reaching the highest number of good CC and RE’s values (Supplementary Figure1.e). The RRGMRES CC’s best results were followed by the ART ones, which were similar than RRGMRES ones (Supplementary Figure1.b), ART-SB and finally, GMRES. However, regarding the RE, the RRGMRES performance was followed by ART-SB, ART and GMRES.

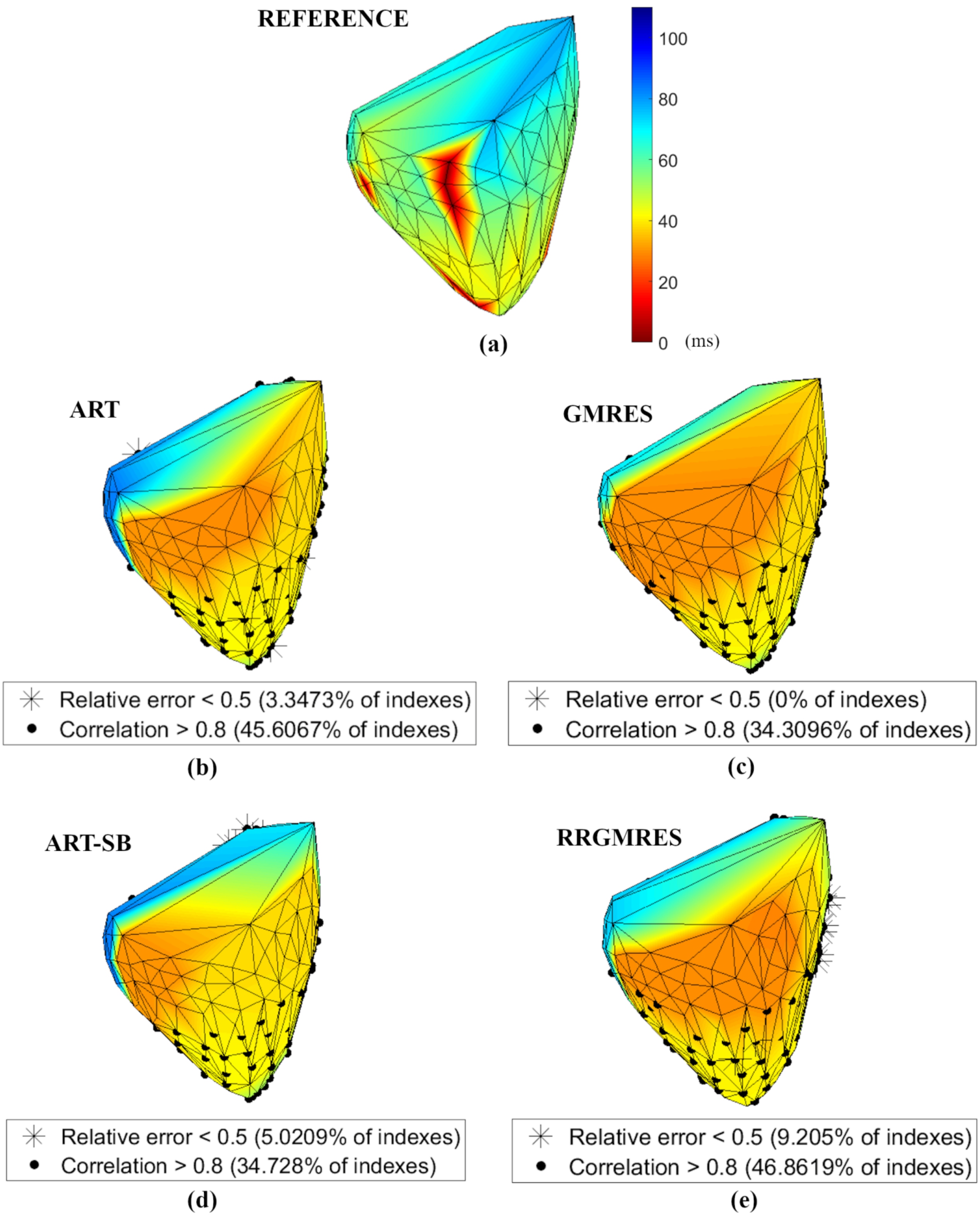

Supplementary Figure 1. Activation maps applied to the sinus rhythm Auckland dataset, for: (a) Reference potentials; (b) ART; (c) GMRES; (d) ART Split Bregman; (e) RRGMRES. All figures

have the same colorbar than (a). For each activation map, the locations corresponding to the "spatial" RE<0.5 and CC>0.8 are overplotted as shown in the legend.

1.2 Supplementary activation maps for Maastricht dataset

Supplementary Figures 2 and 3 shows the dV/dT pattern over activation and recovery time for Maastricht dataset. Additionally, in each figure, we outlined the locations with the best "spatial" CC and RE following the explanation in 2.4.2.

Supplementary Figure 2 showed that, for the sinus rhythm of the Maastricht dataset, the ART achieved the best CCs and REs, since it reached the more points when CC>0.8 and RE<0.5 (Supplementary Figure 2.b). The ART performance was followed by ART-SB and RRGMRES. Here, GMRES returned the worst CCs and REs as it can be observed in Supplementary Figure 2.c.

For the paced rhythm Maastricht dataset, ART-SB presented the best CCs (Supplementary Figure 3.d), followed by RRGMRES and ART (being the last two slowly different). Regarding the RE, ART achieved the best values, followed by RRGMRES. In this case ART-SB and GMRES did not present any reconstructed point with RE<0.5. GMRES performed the worst CC and RE's values (Supplementary Figure 3.c).

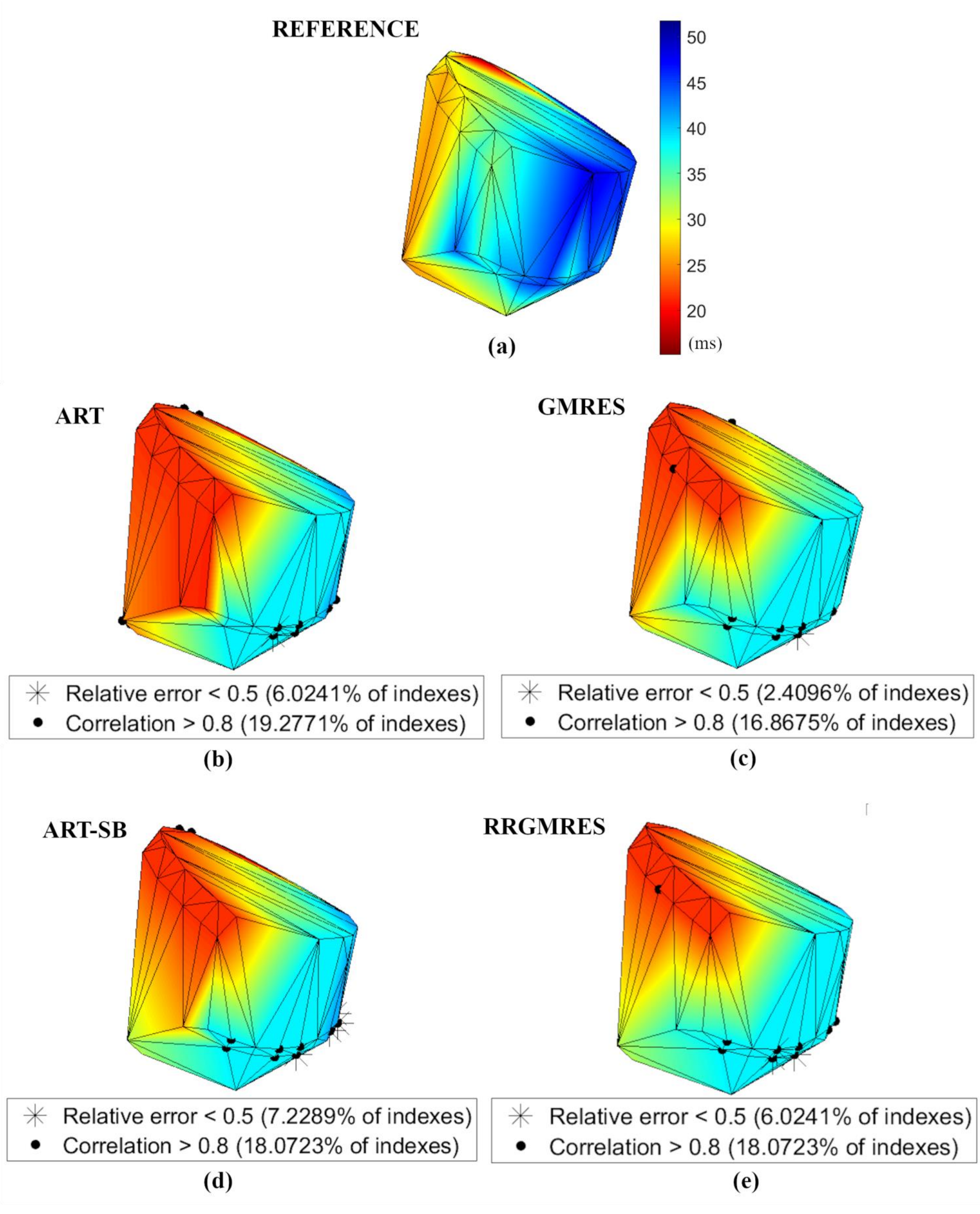

Supplementary Figure 2. Activation maps applied to the sinus rhythm Maastricht dataset, for: (a) Reference potentials; (b) ART; (c) GMRES; (d) ART Split Bregman; (e) RRGMRES. All figures

have the same colorbar than (a). For each activation map, the locations corresponding to the “spatial” RE<0.5 and CC>0.8 are overplotted as shown in the legend.

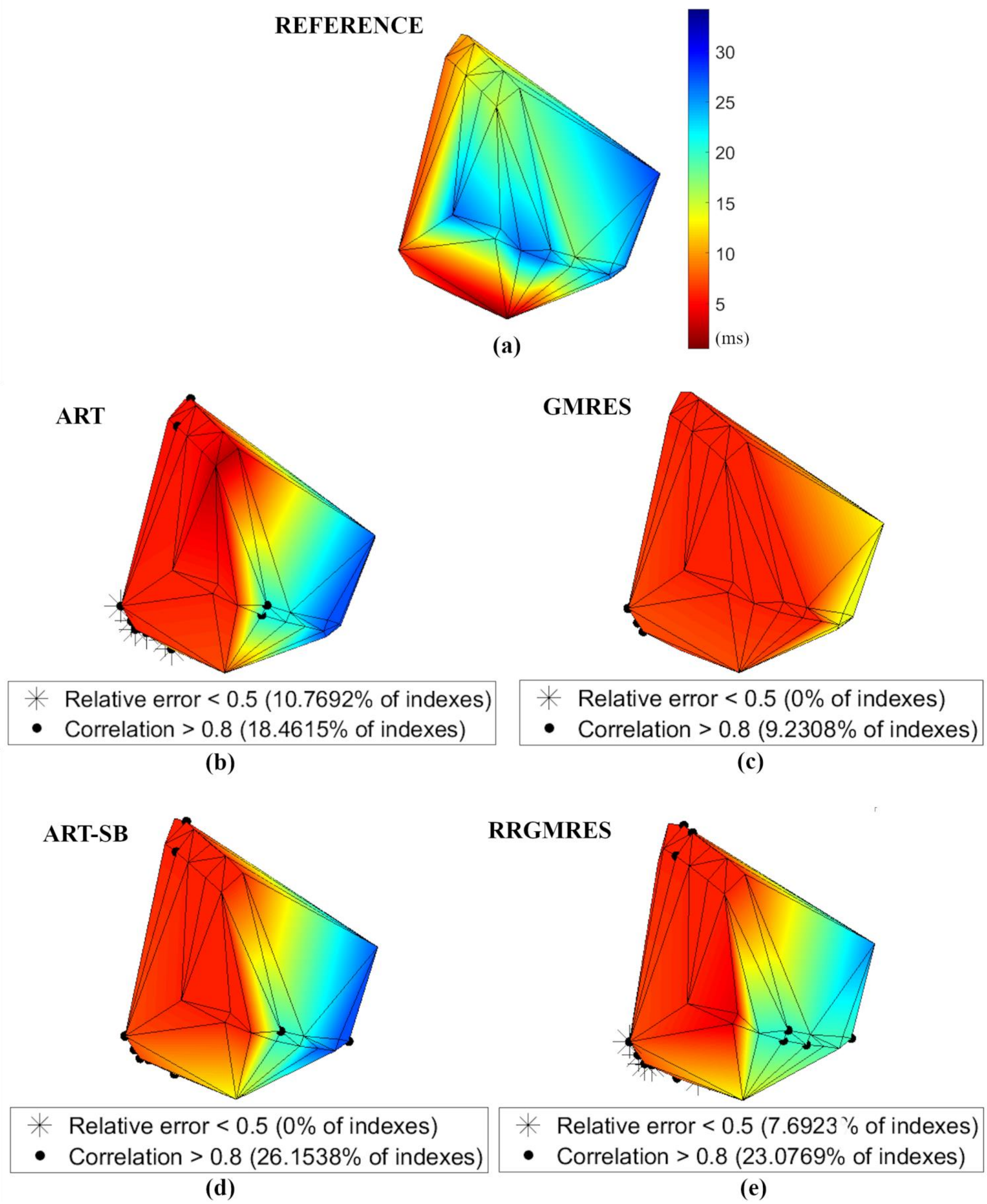

Supplementary Figure 3. Activation maps applied to the paced rhythm Maastricht dataset, for: (a) Reference potentials; (b) ART; (c) GMRES; (d) ART Split Bregman; (e) RRGMRES. All figures

have the same colorbar than (a). For each activation map, the locations corresponding to the “spatial” RE<0.5 and CC>0.8 are overplotted as shown in the legend.

To end, we would like to emphasize that while more reconstruction locations could improve the pacing location in the ventricles, the Supplementary Figure 3 clearly shows a clear improvement of all the proposed iterative methods against the GMRES.
